# Minimally Invasive Transdural Closure of a Ventral Spinal Cerebrospinal Fluid Leak in Spontaneous Intracranial Hypotension: 2-Dimensional Operative Video

**DOI:** 10.1227/ons.0000000000001657

**Published:** 2025-06-10

**Authors:** Levin Häni, Christopher Marvin Jesse, Tomas Dobrocky, Eike Immo Piechowiak, Ralph Thomas Schär, Andreas Raabe

**Affiliations:** * Department of Neurosurgery, Inselspital, Bern University Hospital, University of Bern, Bern, Switzerland;; ‡ University Institute of Diagnostic and Interventional Neuroradiology, Inselspital, Bern University Hospital, University of Bern, Bern, Switzerland

**Keywords:** Spontaneous intracranial hypotension, Spinal cerebrospinal fluid leak, Orthostatic headache

Spontaneous intracranial hypotension (SIH) is caused by spontaneous cerebrospinal fluid (CSF) leaks at level of the spine. Symptoms include orthostatic headache, nonorthostatic headache, and vestibulocochlear complaints.^1^ Dynamic myelography techniques are crucial to pinpoint the site of the leak.^2^ Most leaks occur in the ventral dura (type 1 leak). Other types include a leaking spinal nerve root diverticulum (type 2) or a direct CSF-venous fistula (type 3).^2,3^ We present the case of a 68-year-old male patient with typical symptoms and imaging signs of SIH. Cranial MRI demonstrates signs indicative of a CSF leak.^4^ Spinal MRI including heavily T2-weighted, fat-suppressed sequences demonstrate a spinal longitudinal epidural collection. Dynamic CT myelography demonstrates a ventral spinal leak at the T11/12 level. To improve symptoms and prevent sequelae of untreated SIH, we offered surgical treatment to this patient.^5-10^ Our video presents a minimally invasive transdural approach with the use of a tubular retractor system as previously described.^11^ This approach is a less invasive alternative to open posterior, lateral, or ventral approaches. In addition, the use of the tubular retractor allows a more oblique angle of attack to the ventral dura compared with traditional open posterior approaches. We apply intradural and extradural sandwich patches to the dura, which has been proven noninferior to suturing.^12^ The patient consented to the procedure. As all patient information was anonymized, this operative video did not require Institutional Review Board approval.
